# Expression Status of Rap1 Pathway-Related Genes in Liver Metastases Compared with Corresponding Primary Colorectal Cancer

**DOI:** 10.3390/cancers16010171

**Published:** 2023-12-29

**Authors:** Maryam Abbastabar, Heike Allgayer, Mahdi Sepidarkish, Farzin Sadeghi, Maryam Ghasemi, Roghayeh Pour-bagher, Hadi Parsian

**Affiliations:** 1 Student Research Committee, Babol University of Medical Sciences, Babol 47176-47745, Iran; maryam.atbr1372@gmail.com; 2 Department of Clinical Biochemistry, School of Medicine, Babol University of Medical Sciences, Babol 47176-47745, Iran; 3 Department of Experimental Surgery-Cancer Metastasis, Medical Faculty Mannheim, Ruprecht-Karls-University Heidelberg, 68167 Mannheim, Germany; heike.allgayer@medma.uni-heidelberg.de; 4 Department of Biostatistics and Epidemiology, School of Public Health, Babol University of Medical Sciences, Babol 47176-47745, Iran; mahdi.sepidarkish@gmail.com; 5 Cellular & Molecular Biology Research Center, Health Research Institute, Babol University of Medical Sciences, Ganjafrooz Street, Babol 47176-47745, Iran; sadeghifarzin6@gmail.com (F.S.); r.pourbagherbio@gmail.com (R.P.-b.); 6 Department of Pathology, Immunogenetics Research Center, Mazandaran University of Medical Sciences, Sari 48175-866, Iran; a.zghasemi@yahoo.com

**Keywords:** colorectal cancer, colorectal cancer liver metastasis, NRAS, FGF1, KDR, NGF

## Abstract

**Simple Summary:**

Colorectal cancer liver metastasis (CRLM) is the leading cause of colorectal cancer (CRC)-related deaths and remains poorly understood molecularly. Understanding the specific molecular characteristics of metastasis clearly bears the chance for improving treatment, or even preventing, this phenomenon. In this study, we aimed to investigate the expression status of Rap1 pathway-related genes in non-metastatic CRC patients and metastatic ones, as well as in primary CRCs and paired samples of those patients with liver metastases, which may help to support the definition of specific molecular characteristics or biomarkers of metastases.

**Abstract:**

Understanding molecular networks of CRLM is an ongoing area of research. In this study, paired CRC tissue and adjacent noncancerous tissue from 15 non-metastatic CRC patients and paired CRC tissue and matched liver metastatic tissues from 15 CRLM patients along with their adjacent noncancerous tissues were evaluated. We assessed Rap1 pathway-related genes including NRAS, FGF-1, NGF, and KDR expression by qRT-PCR and their protein status by Western blot. In CRLM patients, NRAS, FGF1, and KDR mRNA and protein were expressed at higher levels in metastatic than in CRC primary tumor and adjacent noncancerous tissue (*p* < 0.05). In non-metastatic patients, NRAS, FGF1, KDR, and NGF gene expression did not differ between CRC primary tumor-and adjacent noncancerous tissue (*p* > 0.05). ROC curve analysis showed a reasonable diagnostic accuracy of NRAS, FGF1, KDR, and FGF for the discrimination of metastatic patients from non- metastatic ones on analysis of their primary tumors. The data suggest that further functional studies on Rap1-related genes’ role in CRLM are needed. In conclusion, the present data broaden our knowledge about specific molecular characteristics of CRLM. An increased understanding of the molecular features of metastasis has the potential to create more successful treatment, or prevention, of metastasis, especially in multimodal primary tumor treatment.

## 1. Introduction

Colorectal cancer (CRC) is the third most commonly diagnosed cancer among men and women worldwide, ranking as high as the second leading cause of cancer-related deaths overall [1]. CRC liver metastasis (CRLM) is a leading cause of CRC-related deaths [2]. Despite advances in prevention, detection, and adjuvant therapy, it is estimated that 50% of individuals diagnosed with CRC ultimately will develop metastatic disease to their liver during their lifetime, which eventually results in death for more than two-thirds of these patients [3,4].

In contrast to the comprehensive investigations that have revealed the pathogenetic mechanisms leading to the formation of primary tumors, the biological basis of metastatic disease remains poorly understood [5]. Chemotherapy has been the first choice for treatment of metastatic CRC patients; however, the side effects of chemotherapy significantly impact the overall quality of life [6]. Following successes with antibodies against epidermal growth factor receptor (EGFR) and vascular endothelial growth factor (VEGF), the prognosis of patients with mCRC has been improved [7]. However, the main challenge for cancer therapy is metastasis, which is due to the lack of understanding of its molecular characteristics [8]. Thus, understanding the cellular and molecular mechanisms involved in the pathogenesis of CRLM may identify innovative targets for required therapy against this deadly biological process [9,10].

As in the majority of other malignant tumors, the metastasis of CRC is a very complicated process that involves multiple signaling pathways and various mechanisms. Ras-associated protein-1 (Rap1) is an important intracellular signaling molecule that controls diverse cellular programs such as cell adhesions, cell–cell junction formation, cellular migration, and cell polarity by regulating the function of integrins and other adhesion molecules in various cell types [11]. Rap1 also regulates MAP kinase activity in a manner that is highly dependent on the context of cell types [12]. Meanwhile, previous studies have linked Rap1 pathway-related genes including neuroblastoma rat sarcoma (NRAS), fibroblast growth factor 1 (FGF-1), kinase insert domain receptor (KDR), and nerve growth factor (NGF) to metastatic progression in CRLM [13,14,15,16,17].

FGF1 and NGF as growth factors are located upstream of Rap1 pathways [18,19]. FGF1, as a member of the FGF family, plays a significant role in modulating endothelial cell migration and proliferation [16]. NRAS, a small GTPase protein, serves as a binary switch in the signal transduction of most growth factor receptors, including EGFRs and other tyrosine kinase receptors [15,20]. KDR is a crucial VEGF receptor mediating signal transduction triggered by VEGF ligands. The interaction between VEGF and KDR results in the activation of downstream signaling pathways, including Rap1, PLCγ-PKC-MEK-MAPK, and PI3K/AKT pathways [21,22,23].

In this study, we aimed to investigate the expression status of NRAS, FGF-1, NGF, and KDR in non-metastatic CRC patients and metastatic ones, as well as in primary CRC and paired samples of the liver metastatic lesions of patients, which may help to support the definition of specific molecular characteristics or biomarkers of metastases. An increased understanding of the molecular features of metastasis has the potential to create more successful treatment or prevention strategies for metastasis.

## 2. Materials and Methods

### 2.1. Patients and Clinical Samples

**Figure 1:**
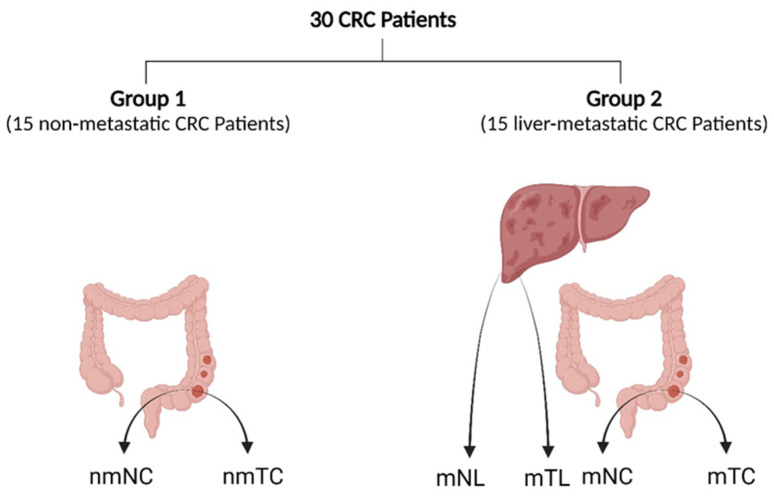
Tissue sampling diagram illustrating the sites of sampling. Abbreviations: nmTC, non-metastatic CRC tissue; nmNC, adjacent noncancerous tissues in non-metastatic patients; mTC; metastatic CRC tissue; mNC, adjacent noncancerous tissues in metastatic patients; mTL, liver metastasis tissue; mNL, adjacent noncancerous tissues in CRLM patients. The figure was created with BioRender.com (accessed on 19 November 2023).

Thirty CRC patients who had undergone surgical resection at the Department of General Surgery, affiliated hospital of Babol University of Medical Sciences, between June 2020 and October 2022 were enrolled in this study. As shown in Figure 1, patients were divided into two groups: Group 1 contains 15 non-metastatic CRC patients (stage I–III) from whom 15 CRC primary tumor tissues (nmTC) and corresponding adjacent noncancerous tissues (nmNC) were collected. Group 2 includes 15 CRLM patients (stage IV) from whom 15 CRC primary tumor tissues (mTC), adjacent noncancerous tissues (mNC), corresponding liver metastasis tissues (mTL), and adjacent noncancerous liver tissues (mNL) were obtained. We restricted our CRLM patients to patients considered to have synchronous metastases, defined as liver metastases detected before or at the time of diagnosis of the primary tumor. In Group 1, all fresh samples were obtained postoperatively and immediately placed into RNA Later solution (cat no. YT9085, Yekta Tajhiz Azma, Tehran, Iran) and stored at −80 °C. In Group 2, formalin-fixed, paraffin-embedded (FFPE) samples were selected based on the quality and representativeness of the sample and were cut into 10 μm thick sections using a microtome (Slee, Nieder-Olm, Germany). FFPE H&E slides from available blocks were reviewed by a pathologist to identify the blocks with a tumor cellular content of at least 75% as an important parameter of quality control. Adjacent noncancerous tissues were resected from tissues over 5 cm away from the tumor edge and confirmed as normal tissues without tumor invasion by postoperative pathology. None of the patients received preoperative radiotherapy, chemotherapy, targeted therapy, or immunotherapy prior to surgery. The tumor stage was classified according to the 7th edition of the AJCC Cancer Staging Manual (2010) [24]. Written informed consent was obtained from all patients and all procedures were approved by the Institutional Research Ethics Committee (IR.MUBABOL.HRI.REC.1401.131).

### 2.2. RNA Isolation from Fresh Specimens

The RNA was extracted from fresh tissues using the FavorPrep Total RNA Mini Kit (cat no. FABRK001, Favorgen Biotech, Pingtung, Taiwan) according to the manufacturer’s protocol. Briefly, after homogenizing the samples in 1 mL YTzol pure RNA (cat no. YT9064, Yekta Tajhiz Azma, Tehran, Iran), 200 μL of chloroform was added to the lysate, shaken vigorously for 30 s, and after 3 min of incubation at room temperature, centrifuged at 12,000× *g* for 10 min at 4 °C. The supernatant was aspirated and 500 µL ethanol (100%) was added. Then, RNA washing was performed using 750 µL of washing buffer (ethanol 80%). Forty microliters of RNase-free ddH2O were added and the samples were centrifuged at 18,000× *g* for 1 min to elute RNA. The purity and concentration of RNA in the samples was measured by nanodrop (Thermo Fisher Scientific, Waltham, MA, USA). Until the final analysis, the RNA samples were stored at −80 °C.

### 2.3. RNA Isolation from FFPE Specimens

Five tissue sections were cut from FFPE blocks and transferred to one microcentrifuge tube per isolation. Deparaffinization was performed with 1 mL xylene for 10 min, twice, followed by washing with 1 mL absolute ethanol for 10 min, twice. Total RNAs were isolated from three air-dried deparaffinized sections per isolation using the FavorPrep Total RNA Mini Kit (cat no. FABRK001, Favorgen Biotech, Pingtung, Taiwan) according to the manufacturer’s protocol. The purity and the concentration of RNA in the samples were measured by nanodrop (Thermo Fisher Scientific, Waltham, MA, USA). Up to the analysis, the RNA samples were stored at −80 °C.

### 2.4. Quantitative Real-Time PCR

RNA samples were transcribed into cDNA using the cDNA Synthesis Kit (cat no. YT4500, Yekta Tajhiz Azma, Tehran, Iran), according to the manufacturer’s instructions. The thermocycling reverse transcription parameters were as follows: 37 °C for 60 min, then 42 °C for 60 min and 70 °C for 5 min. Relative RNA expression levels of NRAS, FGF1, KDR, and NGF genes were analyzed using quantitative real time-polymerase chain reaction (qRT-PCR) on the Magnetic Induction Cycler (Mic) Real-Time PCR System (Bio Molecular Systems, London, England, UK). β-actin was used as a housekeeping gene and control [25,26]. The reaction mixture contained 10 μL 2× SYBR Green qPCR Mix (cat no. YT2551, Yekta Tajhiz Azma, Tehran, Iran), 0.4 μL of each primer specific for the investigated genes (Supplementary Table S1), 7.2 μL of distilled water, and 2 μL of template up to a 20 μL final volume. The qRT-PCR reactions for each sample were performed in triplicates. A negative control without template was also added in duplicate. The real-time PCR conditions were as follows: 10 min, 95 °C for primary denaturation, 40 cycles of 95 °C for 30 s, 58 °C for 1 min, and 72 °C for 1 min. The Pfaffl method was used to calculate relative gene expression data. In subsequent reactions, specific amplification was verified using melting curve analysis.

### 2.5. Western Blotting

Lysates from the tissues and cells were prepared by using RIPA buffer (50 mM Tris-HCl, 150 mM sodium chloride, 0.1% sodium dodecyl sulphate (SDS), 1% NP-40, 0.5% sodium deoxycholic acid, 1 mM sodium orthovanadate, 1 mM NaF, and a protease inhibitor tablet (cat no. 04693116001, Roche, Basel, Switzerland)). The protein concentration of each sample was measured by using the Bicinchoninic acid kit (Pierce, Appleton, WI, USA) and adjusted to a uniform concentration. Thirty micrograms of total proteins from each sample were separated on 10% SDS polyacrylamide gels (SDS-PAGE) and transferred to polyvinylidene fluoride membranes at 100 V for 2.5 h. The membrane was blocked with 5% fat-free milk in Tris-buffered saline and incubated with NGF (sc-518166, Santa Cruz Biotechnology, Dallas, TX, USA) (1:1000), FGF1 (sc-55520, Santa Cruz Biotechnology, USA) (1:1000), KDR (sc-6251, Santa Cruz Biotechnology, USA) (1:1000), and NRAS (sc-31, Santa Cruz Biotechnology, USA) (1:1000) at 4 °C overnight. Then, horseradish peroxidase (HRP)-conjugated goat anti-rabbit IgG secondary antibody (sc-2357, Santa Cruz Biotechnology, USA) (1:5000) was added and incubated for 2 h at room temperature. The results were visualized using chemiluminescence (Millipore, Burlington, MA, USA). Image J software 1.5.4 (National Institutes of Health, Bethesda, Rockville, MD, USA) was used to quantify the protein expression levels, and β-actin (sc-47778, Santa Cruz Biotechnology, USA) (1:300) was used as the control.

### 2.6. Statistical Analysis

Stata version 17 (Stata Corp LP, College Station, TX, USA) software and GraphPad Prism 9.5.1 (GraphPad Software) were used for statistical analysis. The data were presented as the mean with the corresponding 95% confidence interval (CI). For determining normal distribution of the examined variables, we used Q-Q plot, P-P plot, and the Shapiro–Wilk test. The data between two groups were compared using Student’s *t* test, and intra-group comparisons were analyzed using a paired *t* test. Receiver operator characteristic curves and area under the curve (AUC) statistics were used, based on logistic models, to determine the corresponding cutoff points. *p* < 0.05 was considered to be statistically significant for all contrasts.

## 3. Results

The detailed clinical–pathological features of 30 patients are summarized in Table 1.

### 3.1. Expression of NRAS, FGF1, KDR, and NGF mRNA in CRC Tissues

**Figure 2:**
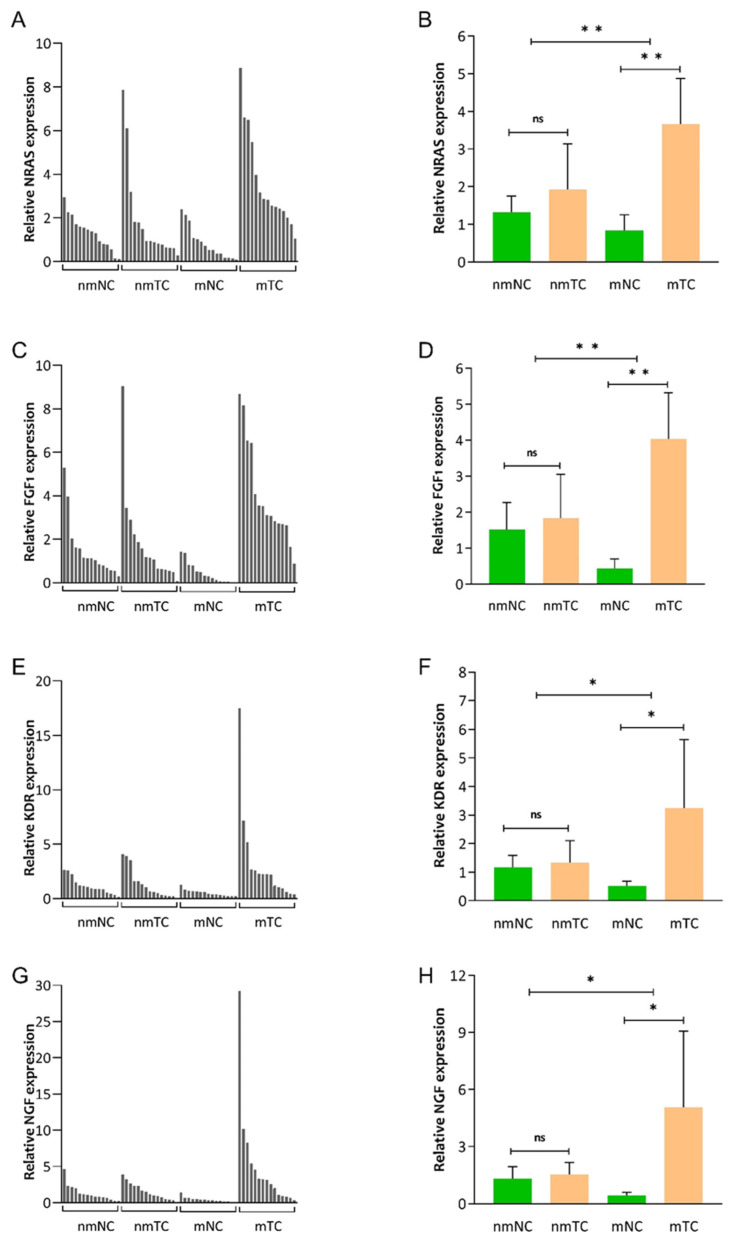
mRNA expression of NRAS (**A**,**B**), FGF1 (**C**,**D**), KDR (**E**,**F**), and NGF (**G**,**H**) in colorectal cancer tissue (nmTC) and corresponding adjacent noncancerous tissues (nmNC) from 15 non-metastatic CRC patients, and in 15 primary CRC tissues (mTC) and adjacent noncancerous tissues (mNC) from 15 CRLM patients. Data are expressed as the mean and 95% confidence interval. ns, not significant (*p* > 0.05); * *p*: 0.001–0.005; ** *p* < 0.001.

To investigate the expression of NRAS, FGF1, KDR, and NGF, we first checked their expression in CRC tissues and compared them with corresponding adjacent noncancerous tissues by qRT-PCR. As shown in Figure 2, in group 2, we found that the mRNA amounts of NRAS (MD: 2.82; 95% CI: 1.46–4.18; *p* < 0.001), FGF1 (MD: 3.60; 95% CI: 2.27–4.93; *p* < 0.001), KDR (MD: 2.72; 95% CI: 031–5.13; *p* = 0.014), and NGF (MD: 4.63; 95% CI: 0.64–8.82; *p* = 0.013) were significantly higher in mTC compared with mNC. Furthermore, in group 1, our results revealed that the mRNA expression of NRAS (MD: 0.60; 95% CI: −0.60–1.81; *p* = 0.149), FGF1 (MD: 0.32; 95% CI: −1.30–1.95; *p* = 0.339), and KDR (MD: 0.16; 95% CI: −0.65–0.99; *p* = 0.335) was not significantly different in nmTC versus nmNC (Supplementary Table S2).

In addition, we analyzed the differences between NRAS, FGF1, KDR, and NGF mRNA levels of CRC tissues in group 1 compared with group 2 patients. Looking at Figure 2, it is clear that the mRNA amounts of NRAS (MD: 2.21; 95% CI: 0.47–3.95; *p* = 0.007), FGF1 (MD: 3.28; 95% CI: 1.27–5.29; *p* = 0.001), KDR (MD: 2.55; 95% CI: 0.12–4.98; *p* = 0.019), and NGF (MD: 4.42; 95% CI: 0.48–8.35; *p* = 0.014) were significantly higher in group 2 compared with group 1.

### 3.2. Expression of NRAS, FGF1, KDR and NGF mRNA in CRLM Patients 

**Figure 3:**
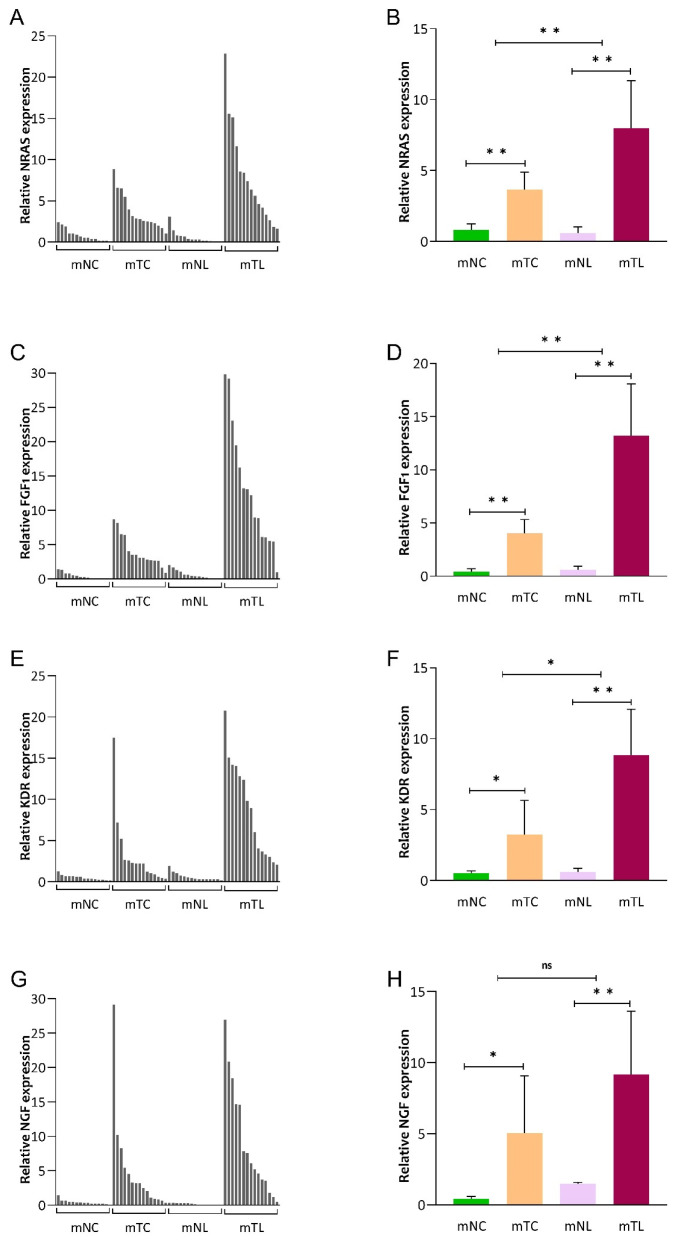
mRNA expression of NRAS (**A**,**B**), FGF1 (**C**,**D**), KDR (**E**,**F**), and NGF (**G**,**H**) in primary CRC tissues (mTC), adjacent noncancerous tissues (mNC), corresponding liver metastasis tissues (mTL), and adjacent noncancerous tissues (mNL) from 15 CRLM patients. Data are expressed as mean (95% CI). ns; not-significant (*p* > 0.05), * *p*: 0.001–0.005, ** *p* < 0.001.

In group 2, we evaluated the mRNA amounts of NRAS, FGF1, KDR, and NGF in mNC, mTC, mNL, and mTL tissues. As shown in Figure 3, we found that mRNA expression of NRAS (MD: 7.40; 95% CI: 4.30–10.51; *p* < 0.001), FGF1 (MD: 12.60; 95% CI: 7.60–17.60; *p* < 0.001), KDR (MD: 8.24; 95% CI: 4.98–11.49; *p* < 0.001), and NGF (MD: 8.99; 95% CI: 4.55–13.42; *p* < 0.001) was significantly higher in mTL compared with mNL (Supplementary Table S2). In addition, NRAS (MD: 4.58; 95% CI: 1.26–7.90; *p* = 0.005), FGF1 (MD: 9.00; 95% CI: 3.64–14.54; *p* = 0.001) and KDR (MD: 5.51; 95% CI: 0.84–10.19; *p* = 0.012) mRNA expression was significantly upregulated in liver metastatic lesions compared with their corresponding primaries. However, our result revealed a higher expression of NGF in liver metastatic lesions in crude data compared with their corresponding primaries even though the difference was not statistically significant (*p* = 0.091) (Supplementary Table S3).

### 3.3. Expression of NRAS, FGF1, KDR, and NGF Protein in CRLM Patients 

**Figure 4:**
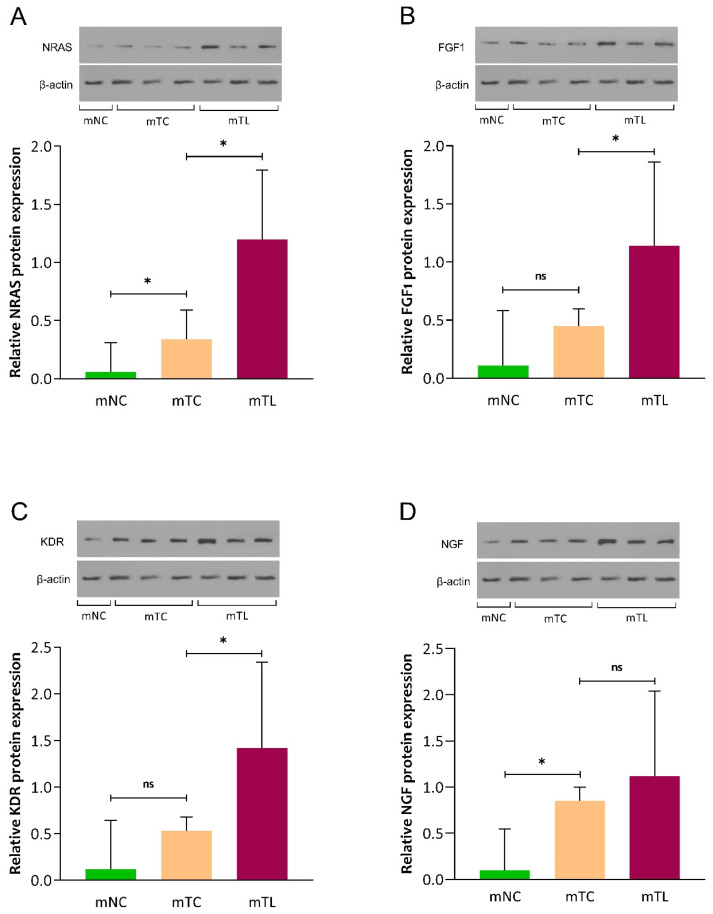
Protein expression of NRAS (**A**), FGF1 (**B**), KDR (**C**), and NGF (**D**) in primary CRC tissues (mTC), adjacent noncancerous tissues (mNC), and corresponding liver metastasis tissues (mTL) from CRLM patients. Data are expressed as mean (95% CI). ns, not-significant (*p* > 0.05); * *p*: 0.001–0.005.

The protein expression of NRAS, FGF1, KDR, and NGF was assessed in mNC, mTC, and mTL tissues of three patients by Western blotting. As shown in Figure 4, we found that the protein expression of NRAS (MD: 0.85; 95% CI: 0.22–1.48; *p* = 0.028), FGF1 (MD: 0.68; 95% CI: 0.08–1.29; *p* = 0.040), and KDR (MD: 0.89; 95% CI: 0.10–1.67; *p* = 0.039 was significantly higher in mTL compared with mTC. Furthermore, our results revealed that the protein expression of NGF was not significantly different in mTL versus mTC (*p* = 0.387) (Table S4 and Figure S1).

### 3.4. ROC Curve Results

**Figure 5:**
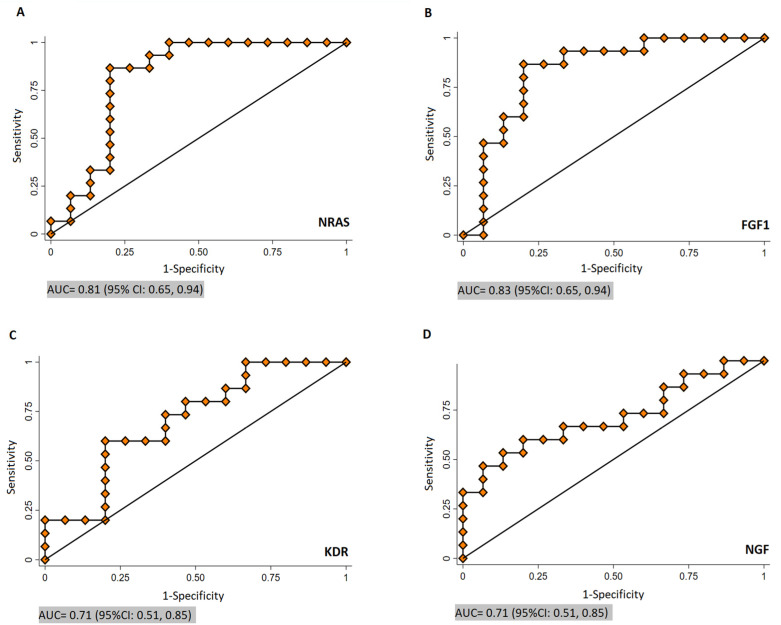
Receiver operating characteristic analysis of mRNA expression of NRAS (**A**), FGF1 (**B**), KDR (**C**), and NGF (**D**). AUC, area under the curve; CI, confidence interval.

The potential of NRAS, FGF1, KDR, and NGF gene expression in discriminating CRLM and non-metastatic CRC patients was evaluated by ROC curve statistics (Figure 5). The diagnostic accuracy of NRAS, FGF1, KDR, and NGF is shown in Table 2.

## 4. Discussion

Despite recent diagnostic and therapeutic advances in cancer, some patients diagnosed with CRC succumb to liver metastatic disease or the complications associated with its treatment [27]. Therefore, understanding specific molecular characteristics of metastases is essential for advancing our knowledge of cancer progression and developing more effective treatment strategies [28]. In this study, we investigated the NRAS, FGF1, KDR, and NGF expression status in liver metastatic lesions with their corresponding primaries, with the rationale to contribute hypotheses to future biomarker development for metastatic CRC.

Recent evidence indicates that there are molecular alterations in primary sites of cancer in metastatic patients compared to non-metastatic patients, as well as metastatic lesions and primary tumors, as a result of molecular variations that improve the potency of metastatic cells to migrate from the primary tumor to the other body sites [29,30,31]. Several studies have recently shown that specific genomic alterations found in mCRC are shared by primary tumors and their matched metastatic samples [32,33], while being absent in non-metastatic CRC [34,35]. Toward this end, Li et al. reported that glucose transporter 1-associated lncRNA was upregulated in CRLM tissues compared with primary CRC tissues or matched normal tissues [36]. In another study, Broker and colleagues reported that two urinary peptides, both parts from collagen type 1 (AGPP(-OH)GEAGKP(-OH)GEQGVP(-OH)GDLGAP(-OH)GP and KGNSGEP(-OH)GAPGSKGDTGAKGEP(-OH)GPVG), could discriminate patients with CRLM from healthy controls [37]. In a large study, Vermaat et al., in targeted exon sequencing of FFPE samples in 21 mCRC patients, clearly saw considerable genomic changes in liver metastases as compared to corresponding primary tumors, which was supported by Kim et al. [38,39].

In recent years, attempts have been made to understand the specific molecular characteristics of metastases at the epigenetic, transcriptomic, and proteomic levels as the “metastasome” [8]. This approach undoubtedly carries that the treatment, or perhaps the prevention, of metastasis might become possible [40]. In a pioneer genome study of metastases of CRC, Ishaque et al. characterized the metastatic lesions of 12 patients, together with their primary tumors and corresponding normal samples. They established evidence that the metastases harbor genomic changes that are specific to the metastases, not being shared with the primary tumor. They pointed out particular genomic lesions, i.e., mutations, within genes such as FGF1 or KDR, which were especially evident in liver metastases compared to corresponding primary CRC tumors [5]. These findings are in accordance with our findings on particular markers of the Rap1 axis and emphasize that factors such as NGF, FGF1, KDR, and NRAS expression, and potential molecular alterations, might be interesting to follow as potential biomarkers for metastatic CRC in future studies. We found that NRAS, FGF1, and KDR expression was significantly upregulated in liver metastatic lesions compared with matched primary tumors. Contrary to expectations, this study did not find a significant difference in NGF expression between liver metastatic lesions and matched primary tumors. A possible explanation for this might be related to metastatic recurrence, as the metastatic cells could affect the primary tumor growth [41]. Another possible explanation for this is sampling variations within a rather small sample size, which, unfortunately, is a well-known fact that cannot be changed since it is extremely difficult to collect large numbers of metastasis tissue samples of patients at one single center. However, the still-significant increase in the expression of this gene in metastatic patients compared to non-metastatic patients indicates a potentially interesting role of this gene in metastatic processes. In the literature, a high expression of NGF was associated with high incidence of metastasis [14]. The mechanism behind this has been suggested to be a binding of NGF to TrkA, leading to phosphorylated TrkA, which activates Rap1, PI3K, and MAPK signaling, promoting CRC metastasis [14].

**Figure 6:**
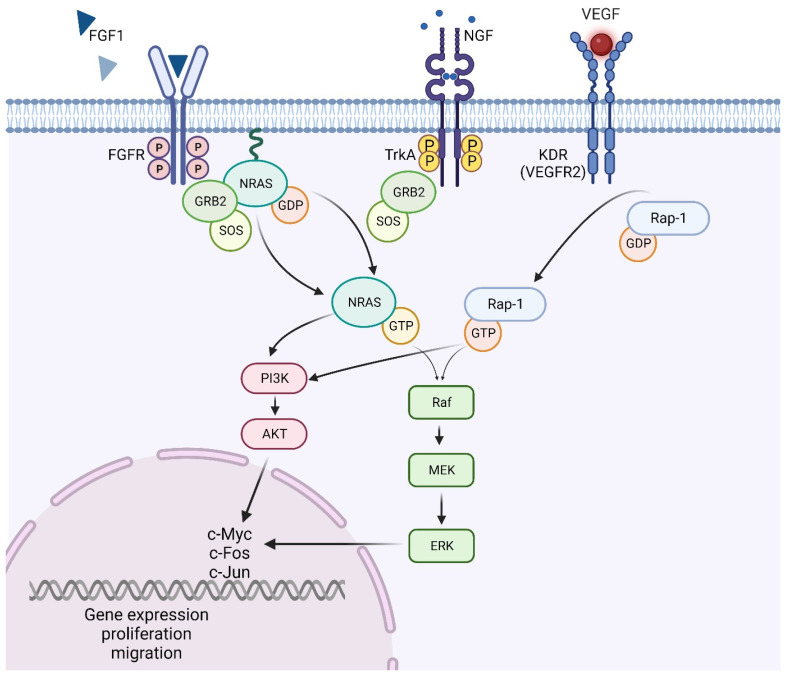
A schematic representation of the Rap1 signaling pathway. The growth factors FGF1, NGF, and VEGF act as external signals which bind to their receptors (FGFR, TrkA, and KDR) located in the cell membrane. This promotes autophosphorylation of the tyrosine domains within the cytosolic site of receptors. These domains are then bound by an adaptor protein, growth factor receptor-bound protein (GRB), which in turn binds to the son of sevenless (SOS) protein. The activated SOS protein will now promote the exchange of GDP, bound to the NRAS protein, to GTP. Activated NRAS will result in the subsequent activation of the downstream signal components of MEK/ERK and PI3K/Akt pathway proteins. Ultimately, this will result in the activation of transcription factors such as Myc, Fos, and Jun in the nucleus, resulting in the regulation of gene expression. The figure was created with BioRender.com (accessed on 19 November 2023) [36].

KEGG pathway analysis showed that FGF1, NGF, KDR, and NRAS genes belong to the Rap1 signaling pathway (Figure 6). The process of CRC metastasis is very complicated and involves numerous signal pathways and various mechanisms [25]. A recent study has reconfirmed that Rap1 signaling is one of the major pathways that regulates the migration and metastasis of cancer cells [42]. Dysregulation of Rap1 pathway-related genes in CRC has been proven. It has been demonstrated that FGF1 is elevated in CRC tissues and induces the invasion and metastasis of tumor cells [16]. In recent years, much attention has been paid to NRAS gene mutations in CRC patients because of their prognostic and predictive roles [17]. Approximately 3–5% of patients with CRLM have mutations within the NRAS gene [17]. A recent case–control study revealed that high expression of NRAS can increase the risk of CRC death up to four times (HR = 4.12, *p* = 0.045) [43]. In addition, Rezaei et al. found that KDR was expressed at significantly higher levels in CRC tissues than in adjacent noncancerous tissues [13]. However, one major drawback of their study was that they were not able to discriminate between metastatic and non-metastatic patients. We found that KDR was significantly higher in the primary CRC tissue of mCRC patients compared to adjacent noncancerous tissues. In contrast, when comparing gene expression in primary CRC tissues and adjacent noncancerous tissues, no statistically significant difference between the mRNA level of KDR in non-metastatic patients was found, implicating KDR as an interesting molecule to study further in the specific context of metastasis.

Our results indicate that Rap1 signaling-related genes are dysregulated in CRLM, providing further support for the hypothesis that they are important for metastasis; it remains to be studied functionally whether they are even relevant for site-specific metastasis to the liver. Based on the classical assumption that site-specific metastasis is activated in an interplay with cells of the target tissue to increase the cancer cells’ capacity to establish metastasis in selected organs, recent efforts already have started to attempt to define the molecular players that contribute to liver tissue tropism in CRC [5]. For example, Ishaque et al. suggested that metastasized cells invoke a response in liver stellate cell pathways that might foster organ-specific metastatic colonization. Furthermore, by functional annotation clustering analysis, it was shown that metastases were notably enriched with extracellular matrix, PI3K–Akt signaling, and pathways associated with focal adhesion [5]. With these issues in mind, it is important to understand the specific molecular characteristics of metastases that have the potential to create more successful treatment or prevention of (site-specific) metastasis.

The early detection of CRLM enhances patients’ chances of effective treatment and improves their prognosis and survival rate [44]. Even though the sensitivity of current imaging diagnosis is still insufficient to achieve early diagnosis, the diagnosis of CRLM still depends on macroscopic imaging examination [45,46,47]. In a recent study, it was found that two panels of micro RNAs (miRNA) could be used for the diagnosis of Stage IV CRC (miR-21 and miR-210) with an AUC of 0.731 and diagnostic accuracy of 69%, and for liver metastasis (miR-210 and miR-203) with an AUC = 0.833 and diagnostic accuracy of 72% [48]. Interestingly, in a first large-scale miR-profiling paper suggesting the metastatically relevant microRNA landscape in CRC and in an early functional paper on miR-21 [49,50], the Allgayer department has already implicated miR-210 and miR-21 as highly relevant for different steps of the CRC metastatic cascade. According to the ROC analysis in our paper, the diagnostic accuracy of NRAS, FGF1, KDR, and NGF for discriminating metastatic CRC patients from non-metastatic CRC patients had a reasonable AUC, specificity, and sensitivity. Still, certainly, the present study has limitations. The sample size was not large and there was some weakness in identifying the cause–effect relationship. Also, in this study, patients with colon and rectal cancer were considered as “CRC”. One drawback is that colon and rectal cancers have distinct anatomical locations and molecular characteristics. By grouping them together, potential differences in disease progression and patient outcomes may be obscured. Still, we are aware of the fact that it is extremely difficult to collect even small numbers of patient metastasis tissues in one single center, so we think that our study still has merits in this regard. Despite its limitations, this study can broaden our knowledge about specific molecular characteristics of CRLM. An increased understanding of these and further molecular features of metastasis has the potential to create more successful treatment or prevention strategies and biomarkers for metastasis.

## 5. Conclusions

This finding implies that Rap1 pathway-related genes are associated with CRC metastasis, their expression being significantly different between CRLM and non-metastatic CRC patients. In addition, the level of gene expression increased in liver metastasis tissue compared to primary CRC and adjacent noncancerous tissues. These data certainly add to our understanding of the specific molecular characteristics of metastases and underscores the selection of specific markers for further CRC metastasis work.

## Figures and Tables

**Table 1:** Clinical characteristics of 30 patients.

Clinical Characteristics		Group 1 (*n* = 15)		Group 2 (*n* = 15)	
		*n*	%	*n*	%
Age at Surgery (year)	≤60	5	33.4	10	66.6
	>60	10	66.6	5	33.4
Gender	male	9	60	11	73.4
	female	6	40	4	26.7
Serum Albumin (g/dL)	normal: 3.5–5.4	13	86.7	12	80
	low: <3.5	2	13.3	3	20
Total Bilirubin (mg/dL)	normal: <2	10	66.6	11	73.3
	high: >2	5	33.4	4	26.7
Tumor Size	≤5 cm	11	73.3	4	26.7
	>5 cm	4	26.7	11	73.4
Stage	I–III	15	100	0	0
	IV	0	0	15	100
pT	T 1–2	9	60	0	0
	T 3–4	6	40	15	100
pN	N0	14	93.4	1	6.6
	N1	1	6.6	14	93.4
M	M0	15	100	0	0
	M1	0	0	15	100

primary tumor (pT), regional lymph nodes (pN), distant metastasis (M).

**Table 2:** The diagnostic accuracy of NRAS, FGF1, KDR and NGF.

Genes	NRAS	FGF1	KDR	NGF
Cut-off point	1.91	2.43	2	1.89
AUC	0.81	0.83	0.71	0.71
*p*-value	0.003	0.001	0.051	0.044
95% CI	0.65–0.94	0.65–0.94	0.51–0.85	0.51–0.85
Sensitivity(%)	86.67	86.67	60	66.67
Specificity(%)	80	80	80	66.67
LR^+^	4.33	4.33	3	2
LR^−^	0.16	0.16	0.50	0.49

Footprint: AUC, Area Under the Curve; CI, Confidence Interval; LR^+^, Positive Likelihood Ratio; LR^−^, Negative Likelihood Ratio.

## Data Availability

The datasets applied and/or analyzed in the current research are available from the corresponding author on reasonable request.

## Supplementary Materials

The following supporting information can be downloaded at: https://www.mdpi.com/article/10.3390/cancers16010171/s1. Table S1: primers used in the study; Table S2: Gene expression status of NRAS, FGF1, KDR, and NGF in non-metastatic CRC patients and liver metastatic CRC patients; Table S3: Gene expression status of NRAS, FGF1, KDR, and NGF in liver metastatic lesions compared with their corresponding primaries of metastatic CRC patients; Table S4: Protein expression status of NRAS, FGF1, KDR, and NGF in liver metastatic lesions compared with their corresponding primaries of metastatic CRC patients; Figure S1: Uncropped Western blot images.
