# Supplementary material for: Expression Status of Rap1 Pathway-Related Genes in Liver Metastases Compared with Corresponding Primary Colorectal Cancer
Source: Cancers (Basel). 2023 Dec 29;16(1):171. doi: 10.3390/cancers16010171 (PMC10778515; doi:10.3390/cancers16010171)
Supplement: Supplementary file 1 [file cancers-16-00171-s001.zip › cancers-2707042-supplementary.pdf]

Supplementary Materials: Expression Status of Rap1 Pathway-Related Genes in Liver Metastases Compared with Corresponding Primary Colorectal Cancer

Maryam Abbastabar , Heike Allgayer , Mahdi Sepidarkish, Farzin Sadeghi, Maryam Ghasemi, Roghayeh Pourbagher and Hadi Parsian

Table S1: Primers Used in the Study

Primer Set	Direction	Sequence (5' → 3')
β-actin	Forward	AGAAAATCTGGCACCACACC
	Reverse	CCATCTCTTGCTCGAAGTCC
NGF	Forward	AGGCTTTGCCAAGGACG
	Reverse	CCAGTGGGCTTCAGGGA
FGF1	Forward	GTGGATGGGACAAGGGACAG
	Reverse	GGCAGGGGGAGAAACAAGAT
KDR	Forward	CTGGCATGGTCTTCTGTGAAG
	Reverse	AATACCAGTGGATGTGATGCGG
NRAS	Forward	CAGAGGCAGTGGAGCTTGA
	Reverse	GCTTTTCCCAACACCACCT

Table S2: Gene Expression Status of NRAS, FGF1, KDR and NGF in Non-metastatic CRC patients and liver metastatic CRC patients

Group		Variable	Mean	SE	SD	95% CI		P-value
						Lower limit	Upper limit	
1 (Non-metastatic CRC patients)	NRAS	nmTC	1.92	0.56	2.20	0.70	3.14	0.14
		nmNC	1.31	0.20	0.78	0.88	1.75	
		Mean difference	0.60	0.56	2.18	-0.60	1.81	
	FGF1	nmTC	1.83	0.57	2.20	0.60	3.05	0.33
		nmNC	1.51	0.35	1.37	0.75	2.26	
		Mean difference	0.32	0.76	2.94	-1.30	1.95	
	KDR	nmTC	1.33	0.35	1.39	0.55	2.10	0.33
		nmNC	1.16	0.19	0.76	0.73	1.58	
		Mean difference	0.16	0.38	1.49	-0.65	0.99	
	NGF	nmTC	1.53	0.29	1.13	0.90	2.15	0.33
		nmNC	1.32	0.29	1.12	0.69	1.94	
		Mean difference	0.20	0.47	1.84	-0.81	1.23	
2 Liver metastatic CRC patients	NRAS	mTC	3.66	0.56	2.20	2.44	4.88	< 0.001
		mNC	0.83	0.19	0.75	0.42	1.25	
		Mean difference	2.82	0.63	2.45	1.46	4.18	
		mTC	7.99	1.55	6.01	4.66	11.32	< 0.001
		mNC	0.59	0.20	0.79	0.15	1.03	
		Mean difference	7.04	1.44	5.60	4.03	10.51	
	FGF1	mTC	4.03	0.60	2.32	2.75	5.32	< 0.001
		mNC	0.43	0.12	0.47	0.17	0.69	

	Mean difference	3.60	0.61	2.40	2.27	4.93	
	mTC	13.21	2.27	8.79	8.34	18.08	< 0.001
	mNC	0.60	0.16	0.63	0.25	0.95	
	Mean difference	12.60	6.59	9.03	7.60	17.60	
KDR	mTC	3.24	1.12	4.33	0.84	5.64	0.01
	mNC	0.52	.07	0.29	0.36	0.68	
	Mean difference	2.72	1.12	4.34	0.31	5.13	
	mTC	8.85	1.50	5.83	5.62	12.08	< 0.001
	mNC	0.61	0.12	0.46	0.35	0.87	
	Mean difference	8.24	1.51	5.87	4.98	11.49	
NGF	mTC	5.05	1.87	7.25	1.04	9.07	0.01
	mNC	0.42	0.08	0.33	0.24	0.60	
	Mean difference	4.63	1.86	7.20	0.64	8.62	
	mTC	9.17	2.07	8.04	4.72	13.63	< 0.001
	mNC	0.18	0.03	0.14	0.10	0.26	
	Mean difference	8.99	2.06	8.00	4.55	13.42	

SE : standard error, SD : standard déviation, CI: confidence interval

Table S3: Gene Expression Status of NRAS, FGF1, KDR and NGF in liver metastatic lesions compared with their corresponding primaries of metastatic CRC patients

Variable		Mean	SE	SD	95% CI		P-value
					Lower limit	Upper limit	
NRAS	liver metastatic lesions	7.04	1.44	5.60	4.03	10.51	0.005
	corresponding primaries	2.82	0.63	2.45	1.46	4.18	
	Mean difference	4.58	1.54	5.99	1.26	7.90	
FGF1	liver metastatic lesions	12.60	6.59	9.03	7.60	17.60	0.001
	corresponding primaries	3.60	0.61	2.40	2.27	4.93	
	Mean difference	9.00	6.66	10.00	3.46	14.54	
KDR	liver metastatic lesions	8.24	1.51	5.87	4.98	11.49	0.012
	corresponding primaries	2.72	1.12	4.34	0.31	5.13	
	Mean difference	5.51	2.17	8.44	0.84	10.19	
NGF	liver metastatic lesions	8.99	2.06	8.00	4.55	13.42	0.091
	corresponding primaries	4.63	1.86	7.20	0.64	8.62	
	Mean difference	4.36	3.11	12.06	-2.31	11.04	

SE : standard error, SD : standard déviation, CI: confidence interval

Table S4: Protein Expression Status of NRAS, FGF1, KDR and NGF in liver metastatic lesions compared with their corresponding primaries of metastatic CRC patients

Variable		Mean	SE	SD	95% CI		P-value
					Lower limit	Upper limit	
NRAS	mTL compared with mTC	0.85	0.25	0.14	0.22	1.48	0.028
	mTC compared with mNC	0.28	0.08	0.05	0.06	0.50	0.031
FGF1	mTL compared with mTC	0.68	0.24	0.14	0.08	1.29	0.040

	mTC compared with mNC	0.34	0.25	0.14	-0.28	0.96	0.146
KDR	mTL compared with mTC	0.89	0.31	0.18	0.10	1.67	0.039
	mTC compared with mNC	0.41	0.26	0.15	-0.25	1.07	0.119
NGF	mTL compared with mTC	0.27	0.43	0.25	-0.80	1.36	0.387
	mTC compared with mNC	0.74	0.16	0.09	0.32	1.16	0.017

SE : standard error, SD : standard déviation, CI: confidence interval

Figure S1. Uncropped Western blot images

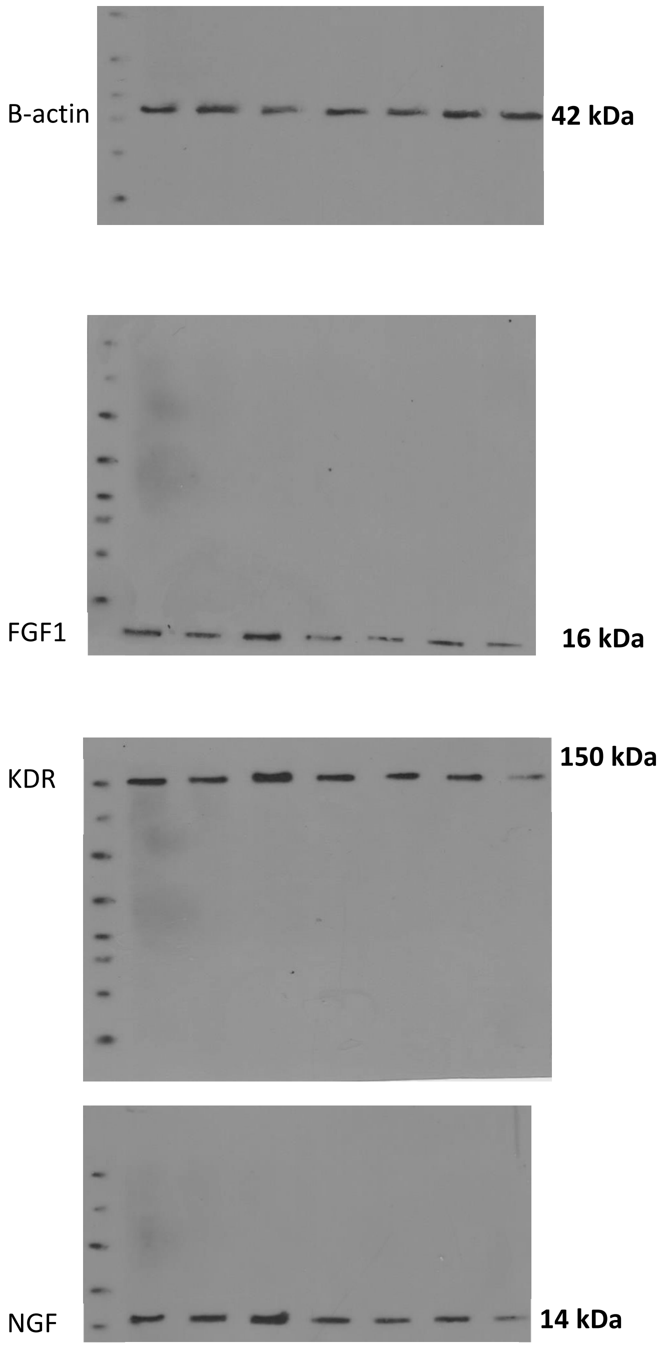

NRAS

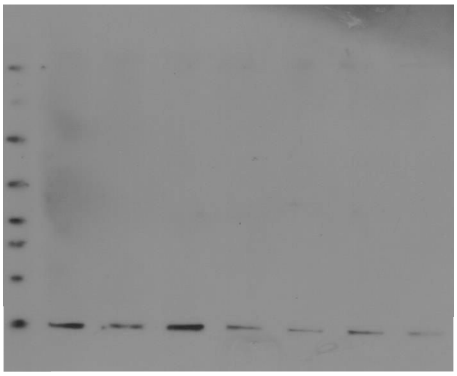

21 kDa
